# Point-of-care detection, characterization, and removal of chocolate bloom using a handheld Raman spectrometer

**DOI:** 10.1038/s41598-020-66820-1

**Published:** 2020-06-17

**Authors:** Joshua Heuler, Siyu He, Sharad Ambardar, Dmitri V. Voronine

**Affiliations:** 1 Department of Cell Biology, Microbiology and Molecular Biology, University of South Florida, Tampa, FL 33620 USA; 2 Department of Biomedical Engineering, Columbia University, New York City, NY 1002 USA; 3 Department of Medical Engineering, University of South Florida, Tampa, FL 33620 USA; 4 Department of Physics, University of South Florida, Tampa, FL 33620 USA

**Keywords:** Optical sensors, Raman spectroscopy

## Abstract

Chocolate bloom is an off-white coating on the surface of chocolate products due to the altered distribution of the ingredients. Bloom reduces the shelf-life of chocolate and affects its visual and tactile quality, all of which are serious concerns for chocolate manufacturers and consumers. The automated, rapid, and noninvasive point-of-care detection of chocolate bloom has been an essential but challenging problem. The ability to detect and characterize chocolate bloom using portable laser spectroscopy could be used to develop *in-situ* quality control sensors. In this work, a handheld Raman spectrometer was used to detect chocolate bloom. Raman spectra acquired from bloomed HERSHEY’S milk chocolate, Hawaiian Host milk chocolate covered macadamia nuts, and Babayevsky Russian dark chocolate were used to characterize the type of bloom. The 1064 nm laser beam of the handheld Raman instrument was used to partially remove the fat bloom of the dark chocolate and to induce sugar bloom on the milk chocolate. The handheld Raman approach has a high potential for industrial and consumer applications for the on-site chemical analysis of chocolate bloom and as an alternative laser-based chocolate decoration.

## Introduction

The main components of milk and dark chocolate include cocoa solids, cocoa butter, and sugar, with milk chocolate containing additional milk solids^1^. The distribution of ingredients in the cocoa butter matrix combined with the crystalline structure of the cocoa are the key determinants of the characteristics of chocolate products^2^. Cocoa butter has six crystalline forms (polymorphs) numbered according to their thermal stability, with polymorph VI being the most stable^3^. Polymorph V is preferred by chocolate manufacturers because it gives chocolate its desirable qualities^4^. The challenge for a chocolate maker is to ensure that their chocolate contains polymorph V when it reaches consumers^5^. One of the greatest limitations on the shelf-life^6^ and overall quality^1^ of chocolate is the formation of chocolate bloom. Chocolate bloom can take on a uniform or a marbled appearance^6^, and the resulting beige appearance on the surface of the milk or dark chocolate is viewed by consumers as an indicator of poor quality^4^. Bloom is classified as fat bloom or sugar bloom based on the chemical composition^7^. However, a combination of both sugar bloom and fat bloom in the same sample has been observed^4^.

Chocolate bloom can be caused by flaws in the chocolate composition (such as fatty fillings^8^), manufacturing errors (such as poor tempering^2^), and improper storage in warm and/or humid conditions^9^. The presence of polymorph VI instead of the polymorph V found in unbloomed chocolate is the main chemical indication of the fat bloom^3^. When chocolate is heated, the cocoa butter polymorphs separate and recrystallize at the surface^4^. Cracks in the chocolate from cooling may accelerate the process^9^. Polymorph VI resists melting during temperature fluctuations, which is thought to produce bloom seed crystals for molten cocoa butter to crystallize around upon cooling^10^. Sugar bloom is caused by a different process. The diffusion of the cocoa alters the distribution of the chocolate ingredients and can trap sugar crystals at the surface^1^. Sugar bloom can also be caused by moisture dissolving sugar and forming crystals at the surface of the chocolate^9^. Either bloom formation renders the product unappealing for consumers and manufacturers.

Detecting and characterizing chocolate bloom rapidly and inexpensively is a serious challenge for the chocolate industry. For example, annual production of more than 3.5 million metric tons of chocolate by over 2000 companies involving over 200,000 people in Europe has been reported, with over 90% of which were small and medium sized enterprises producing chocolates containing fillings, such as hazelnuts, almonds, macadamia nuts, and others^11,12^. The complex heterogeneous composition of pure and filled chocolate products, and a variety of manufacturing conditions lead to a large diversity of the causes of chocolate bloom. In particular, the filled chocolate products are especially susceptible to fat bloom formation via complex oil migration mechanisms^13,14^. The formation of chocolate bloom at the industrial level could happen during the manufacturing and packaging stages and may be due to the variations in the ambient conditions such as temperature and moisture, as well as the chemical compositions of the heterogeneous chocolate products, various fillings, and tempering conditions^15^. Continuous monitoring and quality control of those parameters has been a challenge. Therefore, monitoring the formation of bloom using a Raman spectrometer might be an important manufacturing feedback system, which could prevent manufacturing of the bloomed products at an early stage of the fabrication process. When Raman spectrometers are more widely available at the consumer level, they could also be used to develop an early warning strategy based on the gradually varying ratios of the Raman peaks of fats and sugars. For example, as described below, the laser treatment could amplify bloom formation under certain conditions, which may provide an early warning signal that blooming process has started, potentially, even in the absence of visual effects.

Typically, human panels are used to rate the level of chocolate bloom, but this technique lacks sensitivity and objectivity^6^. Over the past decade, several theoretical and experimental techniques to detect fat bloom such as the whiteness-index^6^ and other mathematical models^16^ have been developed. However, these models require sophisticated calibrations to differentiate between degrees of flat bloom^6^ and have low sensitivity to any changes in the visual description of the chocolate^17^. Cold stage x-ray photoelectron spectroscopy (XPS) was used to experimentally confirm the types of bloom present in chocolate samples based on the observed carbon functional groups^4^. Other techniques have been applied for the purpose of understanding bloom formation. Atomic force microscopy has been used to observe how the blooming process increases the roughness of the chocolate surface^1^. X-ray diffraction was used to differentiate six polymorphs of cocoa butter^3^, and it has been more recently used to observe how temperature changes are related to the presence of the VI polymorph in bloomed chocolate^1,4^.

Raman Spectroscopy is a non-invasive, label-free technique that was previously used for laser-based chemical analysis. Broad applications of Raman spectroscopy include agriculture^18,19^ and the food industry^20^. Raman spectroscopy has also been used to analyze the spectra of cocoa butter polymorphs^21^. Therefore, polymorphs V and VI could be directly discriminated by using Raman spectroscopy. Cocoa butter, a component of milk chocolate^1^, emits a strong Raman signal^21^, but studies of milk chocolate and its constituents using Raman spectroscopy with near infrared (785 nm) and visible (532 nm) lasers were limited by the strong fluorescence background from cocoa solids^22^. Unfortunately, traditional and bulky Raman spectrometers are fixed installations which limit the application of on-site measurements. The use of handheld Raman spectrometers overcomes this limitation and is essential in carrying out mobile Raman measurements. Even in the variable field conditions and environments, portable Raman spectroscopy has been useful in archaeology^23^ and mineralogy^24^ where it was difficult to analyze large archaeological samples using traditional bulky microscopes. Additionally, handheld Raman spectrometers have been applied to art^25^ and rock paintings^26^. Raman spectroscopy has also been used in the field of forensics^27^, such as for the identification of bodily fluids^28^. Therefore, handheld Raman spectroscopy may be considered as a superior technique for the point-of-care chemical chocolate analysis.

In this work, we used a 1064 nm handheld Raman spectrometer to detect and characterize fat and sugar bloom on three different types of chocolates, namely HERSHEY’S milk chocolate, Hawaiian Host milk chocolate covered macadamia nuts, and Babayevsky Russian dark chocolate. The 1064 nm laser reduces the fluorescence background and suppresses the sample damage^29^ from high laser power as compared to 532 nm and 785 nm lasers. In addition to the detection of chocolate bloom, we were also able to partially remove the fat bloom and to induce the sugar bloom on the Babayevsky dark and Hawaiian Host milk chocolate, respectively. Laser-induced bloom could possibly be used as an aesthetic modification of the outer surface of chocolate without modifying the inner composition and taste.

## Materials and Methods

### Materials

The unbloomed chocolate samples were purchased from local supermarkets. The brands of chocolate used were HERSHEY’S milk chocolate (**MC**), Hawaiian Host Island Macs (**HM)**, i.e. milk chocolate with hazelnuts at the center, and Babayevsky Russian dark chocolate (**RC**). Bloomed HERSHEY’S Milk chocolate (**MB**) was obtained by storing a chocolate bar in 23–29 °C ambient air for one week. The bloomed Hawaiian Host chocolate (**HB**) and the bloomed Babayevsky Russian dark chocolate (**RB**) were obtained by storage at a similar temperature range for several weeks before being used in the experiments. For the **RC** and **HM** chocolates, the smooth, dark, and glossy areas were used to obtain the spectra of the unbloomed chocolate, while the white areas were used to obtain the spectra of the bloomed chocolate. All the chocolate products were used within the shelf time before the corresponding expiration dates. A summary of the relevant nutritional information is shown in Table 1.

**Table 1:** Nutritional information for the commercially available MC, HM, and RC chocolate samples from SmartLabel™, Hawaiian Host, and RussianFoodUSA, respectively.

Sample	% Weight_Sugar_	% Weight_Fats_	Ingredients
HERSHEY’S Milk Chocolate (MC)	56.16	31.5	Cane sugar, milk, cocoa solids, cocoa butter, milk fat, lecithin, natural flavor
Hawaiian Host Milk Chocolate (HM)	50	37.5	Cocoa solids, sugar, cocoa butter, milk, soy lecithin, vanillin, dry roasted macadamia nuts
Russian Dark Chocolate (RC)	51.58	36.8	Ground cocoa, sugar, cocoa powder, cocoa oil, emulsifiers

### Instrumentation

**Figure 1:**
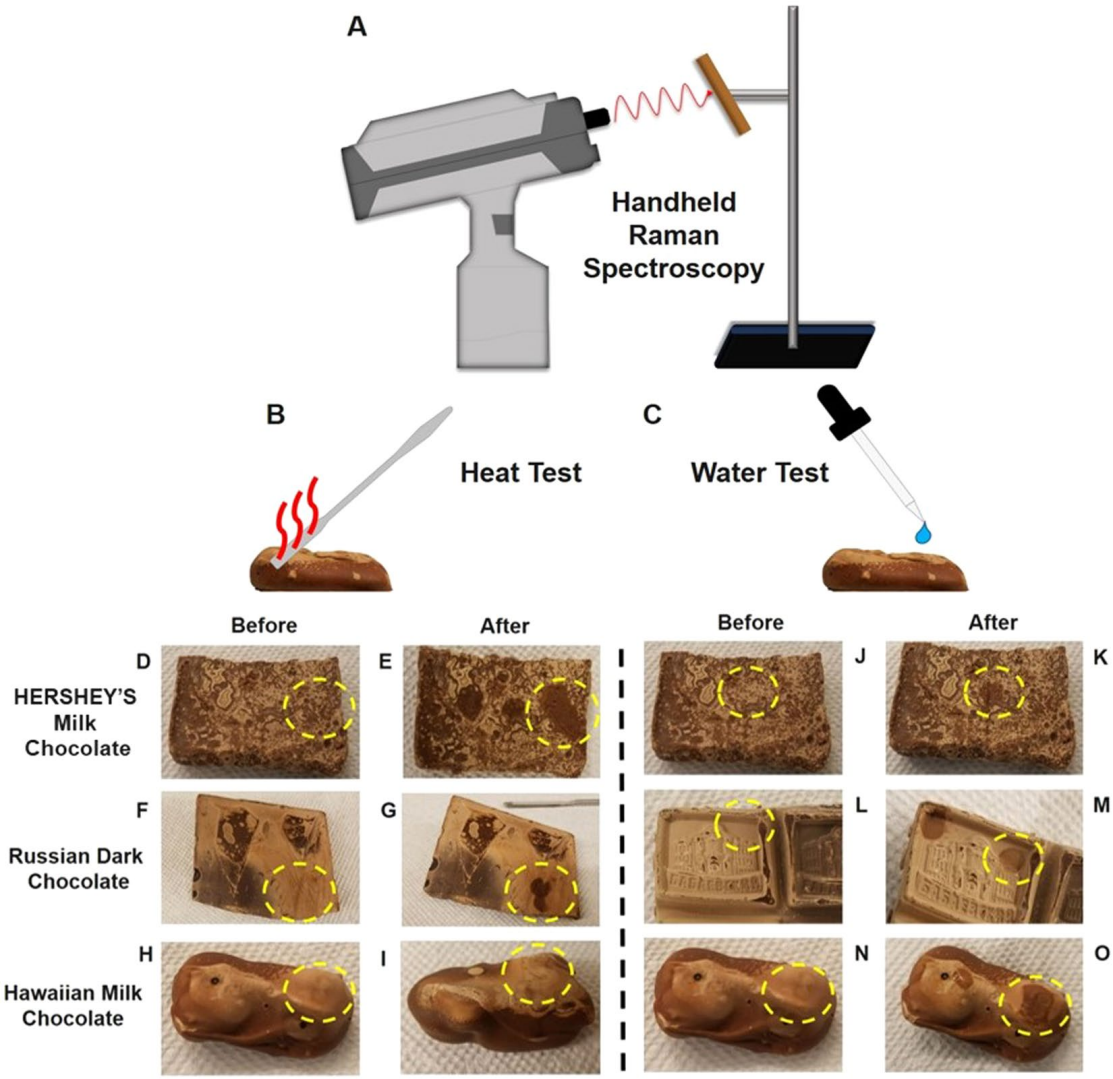
Chocolate bloom analysis. (**A**) Point-and-shoot handheld Raman analysis of a chocolate bar sample fixed on a sample holder and illuminated by a laser beam (red wavy line) from a handheld device. (**B**) Heat test and (**C**) water test are used as control tests to confirm the type of bloom. The yellow dashed circles show the areas of control test application. Optical images of the bloomed chocolate samples before (**D**, **F**, **H**) and after (**E**,**G**,**I**) a hot metal spatula was placed on the bloom surface for 20 seconds. The HERSHEY’S milk chocolate bloom (**MB**) and the Russian dark chocolate bloom (**RB**) both melted under the heat, which is shown as dark coloured spots in (**E**,**G**), respectively. Hawaiian milk chocolate bloom (**HB**) did not melt under these conditions. Optical images of the bloomed chocolate samples before (**J**,**L**,**N**) and after (**K**,**M**,**O**) a drop of water was placed on the surface for two minutes. After removing the water drop from the surface, the **MB** and **HB** were significantly dissolved based on the darker coloration of the spots in (**K**,**O**). The **RB** did not significantly dissolve in water, although a slightly darker spot was observed.

A handheld 1064 nm excitation Raman spectrometer (FirstGuard, Rigaku) was used to obtain the Raman spectra. The schematic arrangement of the experiment is shown in Fig. 1A. The chocolate samples were fixed with clamps on a vertical sample holder, and the handheld Raman instrument was used in point-and-shoot mode to directly illuminate the sample with the laser beam and to collect the scattered Raman signals in the backward direction. The laser point was focused 3 mm from the aperture of the handheld device. For the unbloomed chocolate samples, the laser power was set to 350 mW with 5 s exposure time and 60 accumulations. The laser focal spot was ~1 mm. Some of the bloomed chocolate samples showed melting at 350 mW of power. Therefore, to avoid melting, all the spectra of the bloomed samples were obtained at 300 mW power with the same 5 s exposure time and 60 accumulations. Dark background was taken before every reading and was subtracted from the raw data. The raw spectra were normalized by dividing the intensity of all bands by the highest cocoa butter band (1439 cm^−1^ to 1464 cm^−1^). The corresponding non-normalized spectra are shown in Supplementary Information (Figs. S1, S2 and S5). For measuring the Raman signals from the bloomed chocolate samples, we selected areas of uniform states of bloom formation, which represent mostly bloom with minimal mixture of the unbloomed areas.

### Control Experiments

Two control test experiments were performed to verify the classifications of the bloom samples based on the Raman data. First, in the water test, a drop of water was placed on the bloom surface for two minutes before being removed with a paper towel (Fig. 1C). The dissolved bloom was identified as sugar bloom. Second, the heat test was performed with the end of a metal spatula heated by placing its end on a 40 °C hot plate for 20 seconds. The spatula was then held against the bloom surface for 10 seconds (Fig. 1B). The melted bloom was identified as fat bloom. The results of the invasive control experiments are shown in Fig. 1D–O and are compared to the non-invasive Raman measurements.

## Results and Discussion

### Bloom Detection

#### HERSHEY’S Milk Chocolate

The unbloomed and bloomed chocolate samples were placed in a sample holder shown in Fig. 1A and analyzed with the 1064 nm handheld Raman spectrometer. The Raman bands were assigned to sucrose^22,30^, cocoa butter^21,22,31^ and fats^21,22,31^ as displayed in Table 2. The spectra were normalized by dividing the intensity of all bands by the highest cocoa butter “fat” band (1439 cm^−1^ to 1464 cm^−1^). This and another fats band at 1298 cm^−1^ are mainly due to the –CH_2_ deformation^22,32^. Therefore, the normalized spectra show the ratios of the given band’s assigned constituent and the level of cocoa butter. For the Russian dark and Hawaiian milk chocolate, the dark, glossy areas were used to obtain the spectra of the unbloomed chocolate, while the white areas were used to obtain the spectra of the bloomed chocolate. We optimized the Raman spectral acquisition parameters to obtain high reproducibility and low spectral fluctuations as shown in the two additional **MC** replicate measurements in Supplementary Fig. S6.

**Table 2:** Spectral band assignments.

Band Assignment	MC	MB	RC	RB	HC	HB	Vibrational Mode
Sucrose^22,30^	403 s	403 s	403 s	403 s	403 s	403 s	δ(C9–C3–O2), δ(O14–C4–C3)
	535 s	535 s	535 s	535 s	535 s	535 s	βR_2_(A5)
	645 m	636 s	636 s	636 s	636 m	636 m	β${R}_{1}$(A5)
	847 s	847 s	847 s	847 s	847 s	847 s	$\nu {({\rm{C}}\mbox{--}{\rm{C}})}_{}$
Cocoa Butter^21,22,31^	1055w	1031 m	1039 s	—	1039w	1039 m	${\nu }_{as}{({\rm{C}}\mbox{--}{\rm{C}})}_{T}$
	1079w	1080 m	1080 s	1080 s	1079w	1079w	$\nu {({\rm{C}}\mbox{--}{\rm{C}})}_{G}$
	1120w	1120 m	1120 s	—	1120 m	1120 m	${\nu }_{s}{({\rm{C}}\mbox{--}{\rm{C}})}_{T}$
Fats^21,22,31^	1298 m	—	1298 m	1298 m	1298 m	1298 m	τ (CH_2_)
Cocoa butter^21,22,31^	—	—	1434 m	1343 m	1351 s	1351 s	δ(C–H)
	—	1446 s	1446 s	1446 s	1439 m	—	δ($C{H}_{2}$)
	1454 s	1461w	—	—	1454 s	1454 s	${\delta }_{a}$($C{H}_{3}$)

All spectra acquired for the samples were split into a sugar region (217 cm^−1^ to 1000 cm^−1^) and a fat region (1000 cm^−1^ to 1700 cm^−1^). The following abbreviations were used to indicate the band signal strength: s-strong, m-medium, and w-weak.

**Figure 2:**
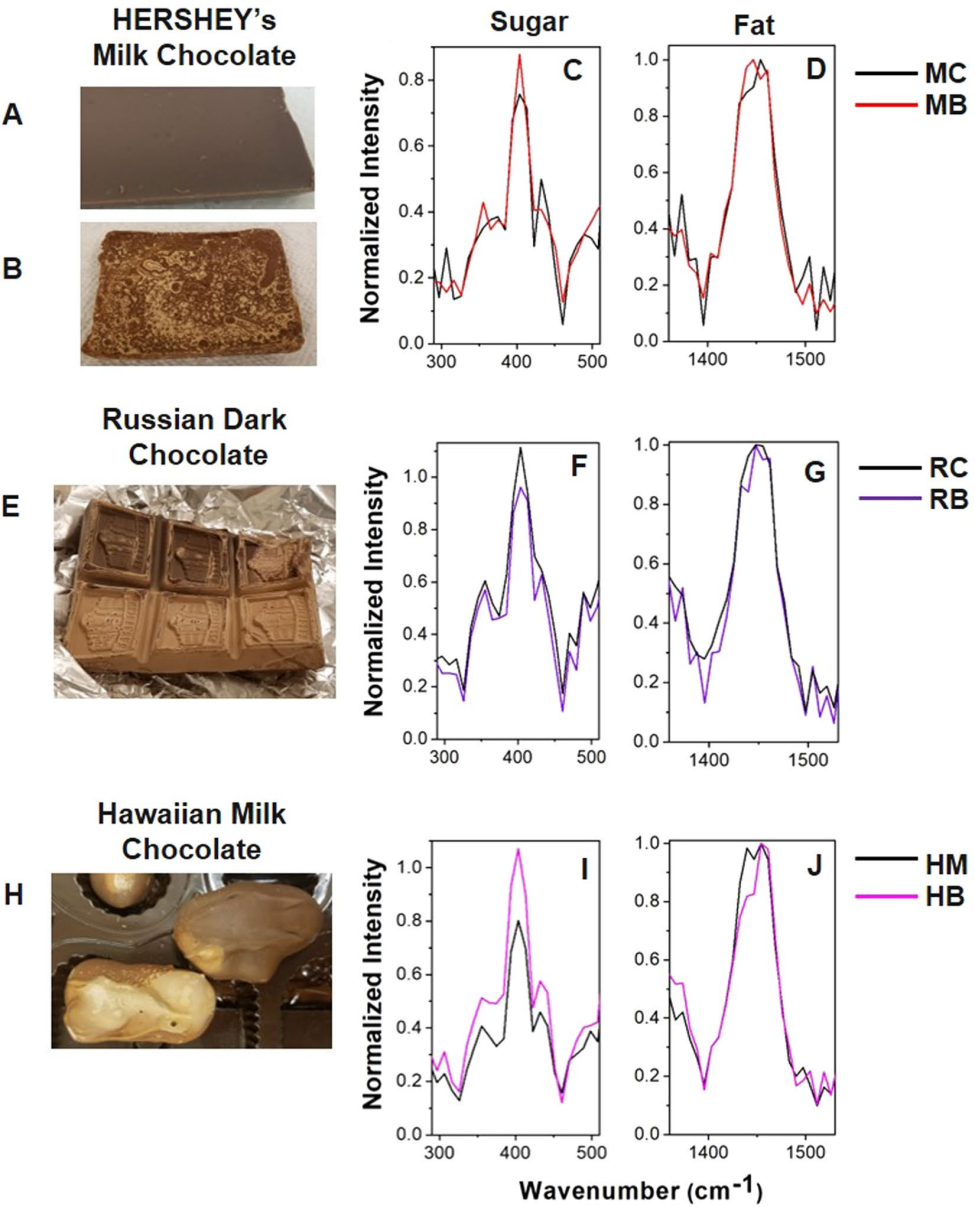
Bloom detection. Optical images of the unbloomed **MC** (**A**), bloomed **MB** (**B**), unbloomed **RC** and bloomed **RB** (**E**), and unbloomed **HM** and bloomed **HB** (**H**) chocolates. The normalized Raman spectra of the sugar (300 cm^−1^ to 500 cm^−1^) and fat (1350 cm^−1^ to 1550 cm^−1^) spectral regions are shown in (**C**,**F**,**I**) and (**D**,**G**,**J**), respectively.

The normalized Raman spectra in Fig. 2C,D show differences between the unbloomed **MC** (black) and bloomed **MB** (red) chocolate samples. The ratio of the sucrose band at 403 cm^−1^ to the cocoa butter band at ~1439–1446 cm^−1^ of the bloomed chocolate is higher than that of the unbloomed sample as shown by the larger intensity of the normalized 403 cm^−1^ in Fig. 2C (the **HB** spectra feature a similar, but significantly greater difference in this band in Fig. 2I). Therefore, the sucrose to cocoa butter ratio is higher in the **MB** chocolate, as the chocolate constituents separate during the blooming process. It is possible that sucrose concentration is increased at the chocolate surface due to the movement of polymorph VI cocoa butter.

Raman spectroscopy suggests that the **MB** chocolate bloom has both sugar and fat components. Fat bloom samples are typically comprised of the cocoa butter polymorph VI, which has a melting point of 34 °C^27^ to 36 °C^3^. Applying heat melts the fat bloom faster than the sugar bloom. On the other hand, the sugar bloom is more water soluble compared to the fat bloom. Figure 1J,K show that placing a water drop on the **MB** chocolate surface dissolves the bloom. Figure 1D,E show that the **MB** bloom melted from the heat of the spatula (the dark regions indicate melted milk chocolate). The results of these control tests indicate that the **MB** bloom is a combination of sugar and fat bloom. Therefore, the control tests corroborate the evidence provided by the handheld Raman spectrometer. Although chocolate bloom is typically classified as either sugar bloom or fat bloom, a mixture of the two has been previously reported^4^. A combination of cryo-scanning electron microscopy, X-ray photoelectron spectroscopy, and environmental scanning electron microscopy was used to detect CH_2_, C-OH, O = C-OH, and O-C-O functional groups on the surface of poorly tempered and untampered chocolates, which indicated the presence of sugar and fat bloom in the same sample^4^. The effects of emulsifiers such as lecithin and flavor ingredients such as vanillin have insignificant contributions to Raman spectra of chocolate due to their small concentrations. For example, the typical concentration of lecithin in chocolate is ~ 0.3–0.6%^11^.

#### Babayevsky Russian Dark Chocolate

In contrast to the HERSHEY’S milk chocolate results, the normalized intensities of the sugar region shown in Fig. 2F are lower in the bloomed **RB** than in the unbloomed **RC** at the 403 cm^−1^ sucrose band. Therefore, the sucrose to cocoa butter ratio is lower in the bloomed **RB** sample. Such changes in the Raman band ratios reflect a decrease in sugars relative to cocoa butter after blooming. This suggests that the blooming process has reduced the amount of sugars at the chocolate surface. Therefore, the Raman data suggests that the Russian dark chocolate bloom is fat bloom. The current bloom theory states that the separation of the polymorph VI of the cocoa butter can displace other ingredients in the chocolate matrix^17^. The evidence from the handheld Raman spectrometer suggests that a change in ingredients at the chocolate surface has occurred.

The control tests shown in Fig. 1 support the fat bloom Raman data. Figure 1M shows that the **RB** bloom surface was not significantly dissolved by a drop of water. This observation is expected because cocoa butter is made of the hydrophobic TAG molecules^33^. Furthermore, Fig. 1F,G show that the bloom surface melts when the heat is applied, which is characteristic of the fat bloom. Therefore, the observed **RB** bloom is fat bloom.

#### Hawaiian Host Milk Chocolate

The ratio of the 403 cm^−1^ sucrose band to the cocoa butter band is significantly stronger in the bloomed **HB** (pink) relative to the unbloomed **HM** (black) as shown in Fig. 2I. Since this data was normalized using the 1454 cm^−1^ cocoa butter band, then the sugar to cocoa butter ratio increased in the **HB** bloom dramatically due to a much higher sugar concentration. As shown in the Supplementary Fig. S1E, the non-normalized intensity of the sugar bands approximately doubled in the bloomed **HB** chocolate.

**Figure 3:**
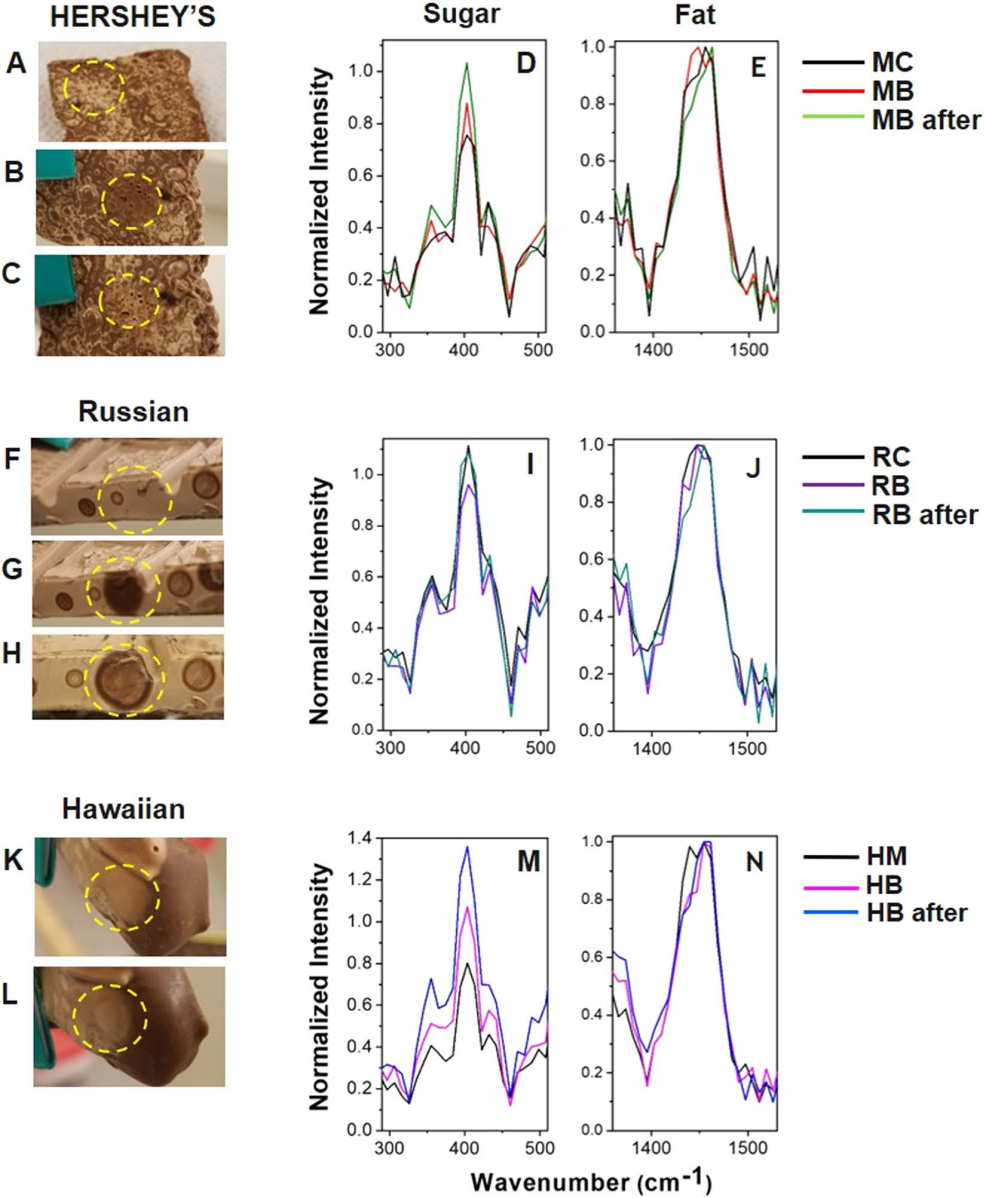
Bloom removal. Optical images of the bloomed chocolate samples before (**A**,**F**,**K**), immediately after (**B**,**G**), and five minutes after (**C**,**H**,**L**) laser treatment. For the **HB** chocolate, the appearance after treatment did not change over time, so only two images are shown. The normalized spectra of the sugar (300 cm^−1^ to 500 cm^−1^) and fat bands (1350 cm^−1^ to 1550 cm^−1^) are shown in the (**D**,**I**,**M**) and (**E**,**J**,**N**) panels, respectively.

There are additional small differences in the fat region of the Raman spectra. The Supplementary Fig. S1F shows that the fat bands increase significantly in intensity after blooming, but they do not quite double in intensity as the sugar bands in Supplementary Fig. S1E. This suggests that the blooming process at the surface of the **HB** may be attributed to sugar bloom. Although the Hawaiian Host sample contained macadamia nuts, there was little evidence to suggest that fat bloom occurred due to the movement of nut oils, which are known to promote fat bloom^34^. If macadamia nut oils moved to the surface, then one would expect the Raman spectra to be dominated by an increase in the relative intensities of the fat bands. However, this was not the case, as the relative intensities of the sugar bands increased significantly more than those of the fat bands. The relative band intensity ratios of the normalized sugar bands of the unbloomed and bloomed samples before and after (see below) the laser treatment are given in Table 3, providing the quantitative analysis of the bloom detection (Fig. 2) and bloom removal (Fig. 3), respectively. The Supplementary Fig. S7 shows non-normalized Raman spectra of all three samples of the unbloomed chocolates in the full spectral range from 217 cm^−1^ to 1800 cm^−1^. The Supplementary Fig. S8 shows the Raman spectra of the MC and MB chocolates for different laser powers, which show the absence of significant differences between the sugar and fat Raman peak ratios.

**Table 3:** Relative band intensity ratios (I) of the normalized sugar bands of the unbloomed and bloomed samples before (left column) and after (right column) the laser treatment.

Sample	${I}_{unbloomed/bloome{d}_{before}}$	${I}_{unbloomed/bloome{d}_{after}}$
HERSHEY’S Milk Chocolate (MC)	0.82	0.67
Hawaiian Host Milk Chocolate (HM)	1.19	1.04
Russian Dark Chocolate (RC)	0.69	0.51

The results of the water drop test support the classification of **HB** as sugar bloom. Figure 1N,O show that the bloom surface is water soluble (the sugar crystals dissolved). Meanwhile, Fig. 1I shows that the bloom surface is resistant to heat. If the bloom were fat-dominated, the surface would have melted and become darker, but Fig. 1H,I show that the surface gets whiter.

### Laser Bloom Removal

#### HERSHEY’S Milk Chocolate Bloom

We further investigated the possibility of laser removal of chocolate bloom and its detection using handheld Raman spectroscopy. The **MB** chocolate revealed visible changes during the laser bloom removal. After the **MB** bloom was exposed to the handheld Raman laser beam for about five minutes, the white color of the bloom decreased, but the chocolate had small holes at the surface (Fig. 3A–C). After five minutes of cooling, Fig. 3C shows that the white color of bloom reappears across the region heated by the laser.

Figure 3D shows that the relative intensity of the 403 cm^−1^ sucrose band increases in the **MB** sample after the laser treatment compared to the original **MC** and **MB** samples. This conveys that the sugar to cocoa butter (fats) ratio has increased, and there is more sugar on the surface. After the laser application melts the surface and the chocolate cools, the cocoa butter may recrystallize at the surface of the chocolate differently from the natural chocolate bloom. Sugar crystals may be moved towards the surface in this process as well. Fat bloom normally forms over a longer time period of melting and recrystallizing of cocoa butter, whereas here the reappearance of the whitish surface happened in a few minutes.

#### Russian Dark Chocolate Bloom

The laser-exposure of the **RB** chocolate bloom temporarily restored the appearance of the dark chocolate in a semi-melted state. The white color of bloom on the **RB** chocolate disappeared entirely with no surface damage (Fig. 3F,G). However, after five minutes a light whitish layer appeared on the surface of the chocolate as shown in Fig. 3H.

The ratio of the 403 cm^−1^ sucrose band to the cocoa butter band in the fat region increased after the laser bloom treatment (Fig. 3I). The changes suggest that the heat of the laser melted the chocolate, and as it cooled the sugars recrystallized in a manner that increased their Raman signals. Unlike the **MB** chocolate, the returning white layer was uneven in the **RB** chocolate. The reforming layers of fat and sugar do not appear to have the same concentrations as either the bloomed or unbloomed samples. This confirms the partial removal of the fat bloom on the Russian dark chocolate. The optical and Raman data also suggest that the **RB** fat bloom was partially converted to sugar bloom.

The practical applications of this laser bloom removal method are currently limited to small areas and short time after the laser treatment, as well as to a specifically fat bloom type. This work presents a promising step toward large scale bloom removal. Optimization of laser wavelength, power, and exposure parameters may further improve the bloom removal efficiency beyond the first proof-of-principle shown in this work.

#### Hawaiian Host Milk Chocolate Bloom

The **HB** chocolate bloom showed a different behavior. Figure 3K,L show that the **HB** bloom has a lighter color after the laser application. Figure 3M shows that the sugar to cocoa butter ratio increased after the treatment. In the Supplementary Figs. S1E,F, the band intensity of the post-treatment spectra is twice as high as the **HB** bloom spectra, which is itself approximately double the intensity of the unbloomed **HM** chocolate. These changes suggest that laser exposure exacerbates the sugar bloom formation rather than reducing it.

### Laser-Induced Bloom

**Figure 4:**
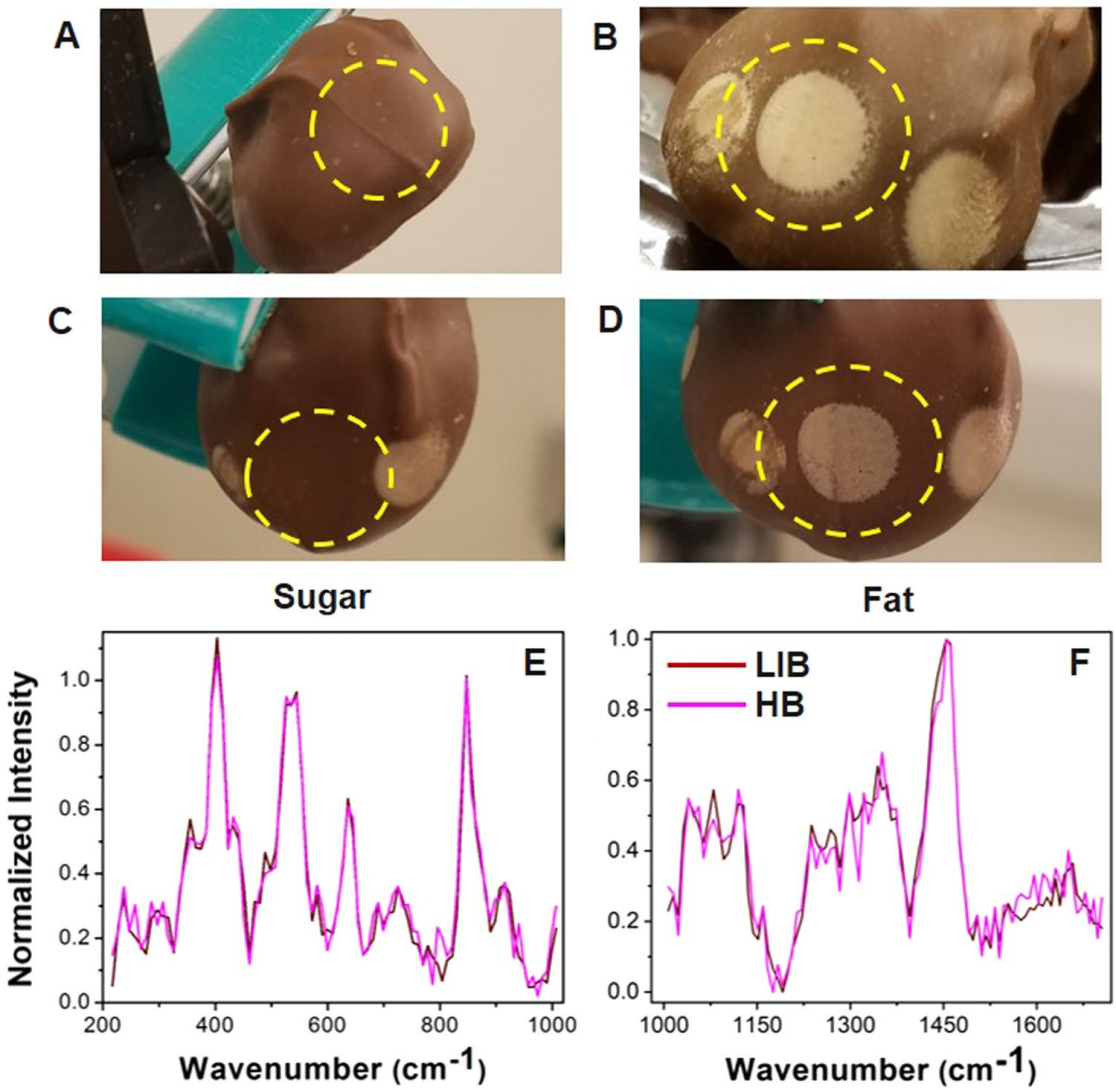
Laser-induced bloom. Optical images of **HM** chocolate before (**A**) and after (**B**) acquiring a Raman spectrum, after the laser was reapplied to the white spot, and melted the bloom (**C**) and after five minutes, the spot reappeared (**D**). Raman spectra of the sugar bloom on the **HB** chocolate and the white spot (LIB) are split into sugar (**E**) and fat (**F**) spectral regions. The two spectra are almost identical, which suggests that the LIB white spot is chemically identical to the **HB** sugar bloom.

The laser-chocolate interaction in the **HM** chocolate (Fig. 4A) resulted in laser-induced bloom (**LIB**) that was not observed in other chocolate samples. After a Raman measurement was taken and the sample cooled for approximately five minutes, a white layer appeared on the surface as a large spot (dashed circle in Fig. 4B). After a second reading was taken on the same spot with the same laser parameters, the spot melted (Fig. 4C) and reformed again after five minutes (Fig. 4D). As shown in the Raman spectra in Fig. 4E,F, the two areas had almost identical signals in terms of band positions and intensities, indicating almost 100% bloom formation. The laser induced bloom happens with a sufficient time delay after the Raman measurement. Therefore, it does not interfere with the process of bloom detection.

When a laser beam was incident on the chocolate, some of the energy was absorbed, and the heat melted the surface. We hypothesize that when the sample cools, the fat and sugar crystallize in an even, white layer. The laser-irradiated spot is, therefore, chemically identical sugar bloom, which is supported by the similar Raman spectra. Laser-induced bloom could be a possible aesthetic etching modification without affecting the bulk of the chocolate.

## Conclusions

Point-of-care handheld 1064 nm Raman spectroscopy was successfully used to detect, characterize, and classify chocolate bloom in milk and dark chocolate. Differences in absolute and relative intensity, band position, and noise levels were used to experimentally confirm the presence of bloom. Control bloom tests verified the assignments. Fat bloom interacted with the laser radiation by melting in the immediate vicinity of the laser spot and then solidifying after a few minutes. The bloom that returned was visually different from the original bloom, and it had stronger Raman signals, which correspond to sugar bloom. Sugar bloom did not melt, and it lightened in color upon the laser treatment. Absolute intensities of the Raman signals increased in the sugar bloom as well. Therefore, laser exposure resulted in the partial removal of fat bloom but did not remove the sugar bloom. Laser exposure induced bloom formation on the surface of **HC** milk chocolate. This could be a method of decorating chocolate products by creating a two-tone effect provided the chocolate is stored in a manner that prevents the spread of bloom. The current cost of a handheld Raman spectrometer limits the wide practical applications for chocolate decorations. However, it is envisioned that cheaper chocolate decoration devices could be built using less expensive lasers without the Raman detection unit.

## Supplementary information

Supplementary Information.
