# Supplementary material for: Point-of-care detection, characterization, and removal of chocolate bloom using a handheld Raman spectrometer
Source: Sci Rep. 2020 Jun 17;10:9833. doi: 10.1038/s41598-020-66820-1 (PMC7299933; doi:10.1038/s41598-020-66820-1)
Supplement: Supplementary file 1 — Supplementary Information. [file 41598_2020_66820_MOESM1_ESM.docx]

Supplementary Information

**Point-of-care detection, characterization, and removal of chocolate bloom using a handheld Raman spectrometer**

Joshua Heuler,^1^ Siyu He,^2^ Sharad Ambardar^3^ and Dmitri V. Voronine^3,4*^

^1^ Department of Cell Biology, Microbiology and Molecular Biology, University of South Florida, Tampa, FL 33620, USA

^2^ Department of Biomedical Engineering, Columbia University, New York City, NY 1002, USA

^3^ Department of Medical Engineering, University of South Florida, Tampa, FL 33620, USA

^4^ Department of Physics, University of South Florida, Tampa, FL 33620, USA


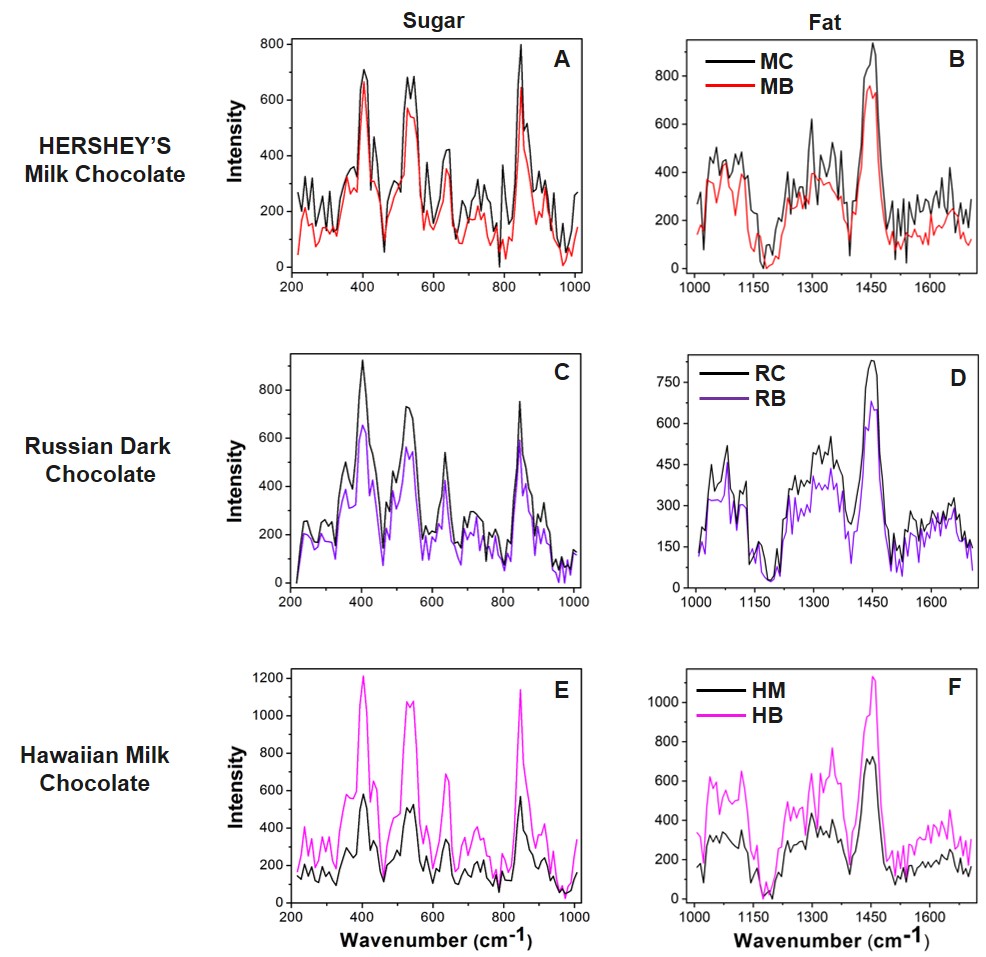


**Supplementary Fig. S1**. Non-normalized Raman spectra of unbloomed and bloomed chocolate. Non-normalized Raman spectra of unbloomed **MC** and bloomed **MB** (**A**, **B**), unbloomed **RC** and bloomed **RB** (**C**, **D**), and unbloomed **HM** and bloomed **HB** (**E**, **F**) chocolates.


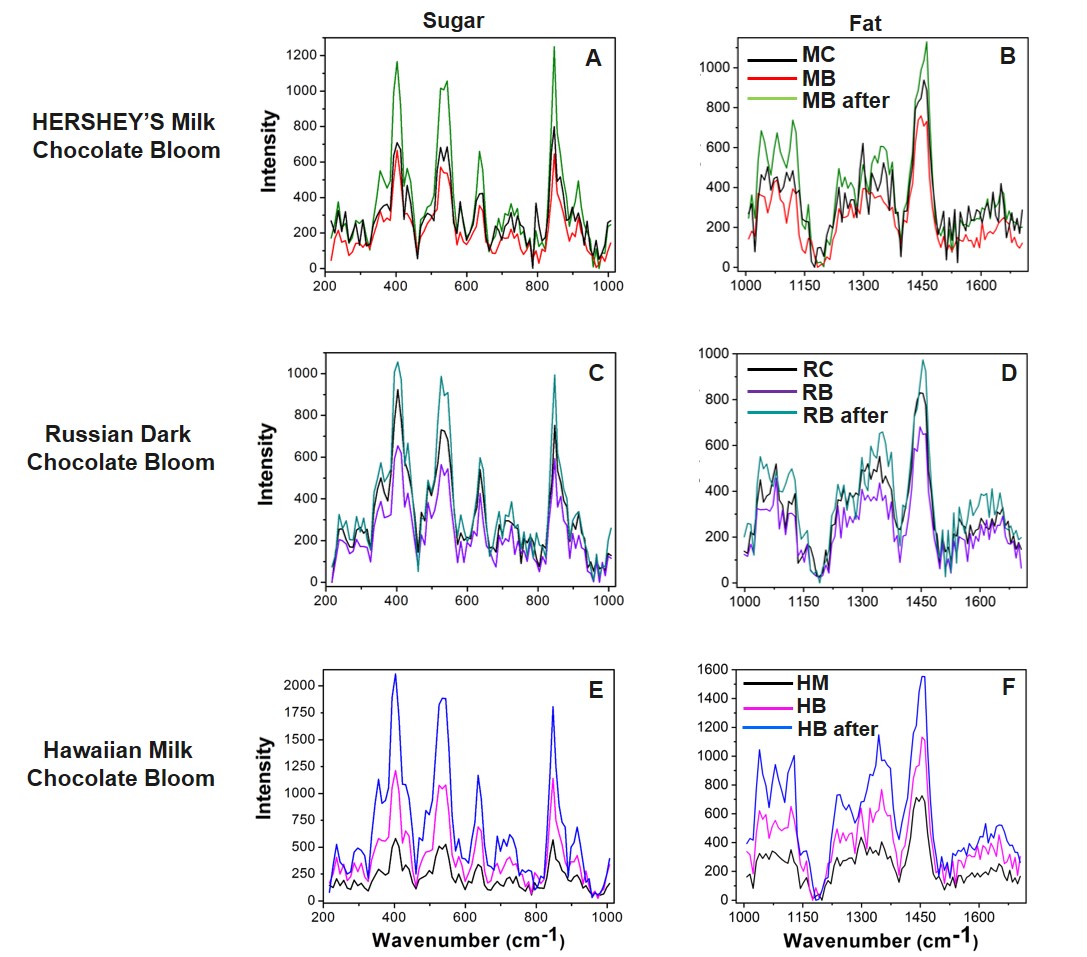


**Supplementary Fig. S2.** Non-normalized Raman spectra of laser bloom removal. The non-normalized Raman spectra of unbloomed **MC** and bloomed **MB** (**A**, **B**), unbloomed **RC** and bloomed **RB** (**C**, **D**), and unbloomed **HM** and bloomed **HB** (**E**, **F**) before and after laser treatment.


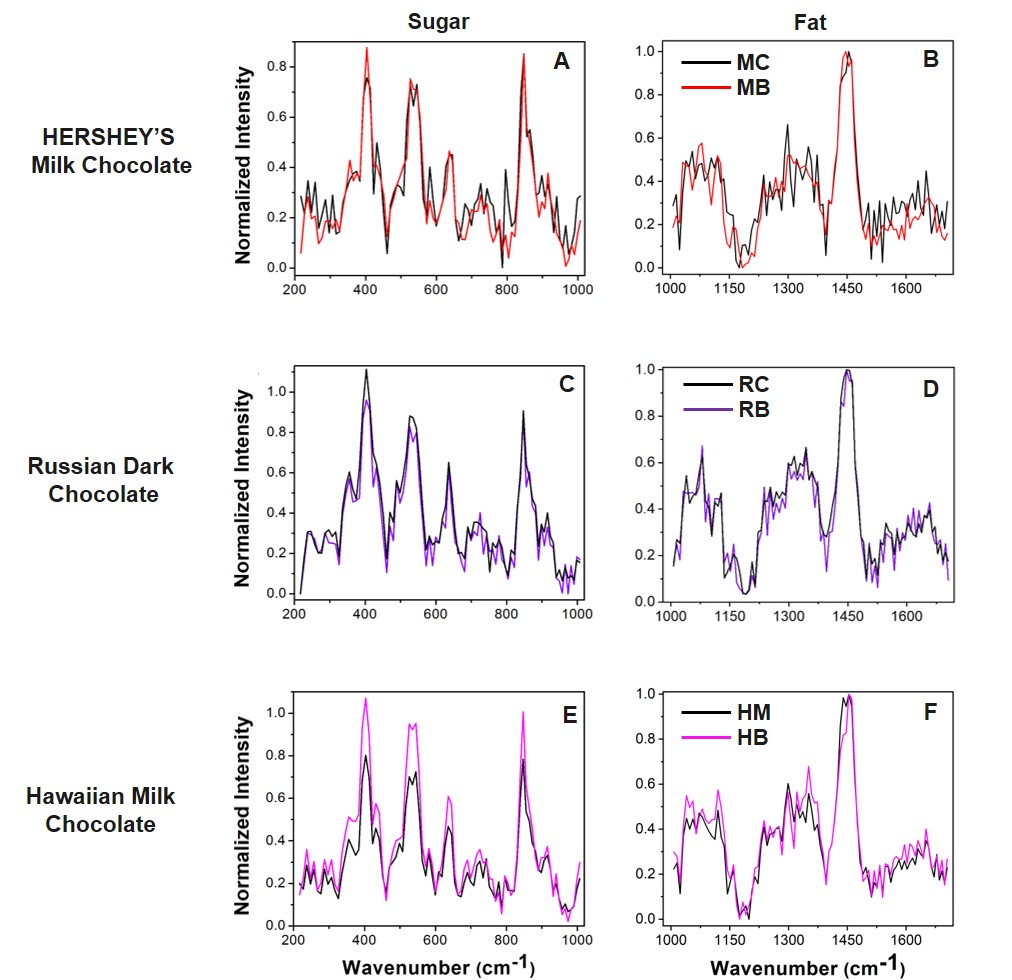


**Supplementary Fig. S3**. Normalized Raman spectra of the sugar (217 cm^-1^ to 1000 cm^-1^) and fat (1000 cm^-1^ to 1700 cm^-1^) spectral regions of bloomed and unbloomed chocolate are shown in (**A, C, E**) and (**B**, **D**, **F**), respectively. The raw spectra were normalized by dividing the intensity of all bands by the highest cocoa butter band (1439 cm^-1^ to 1464 cm^-1^).


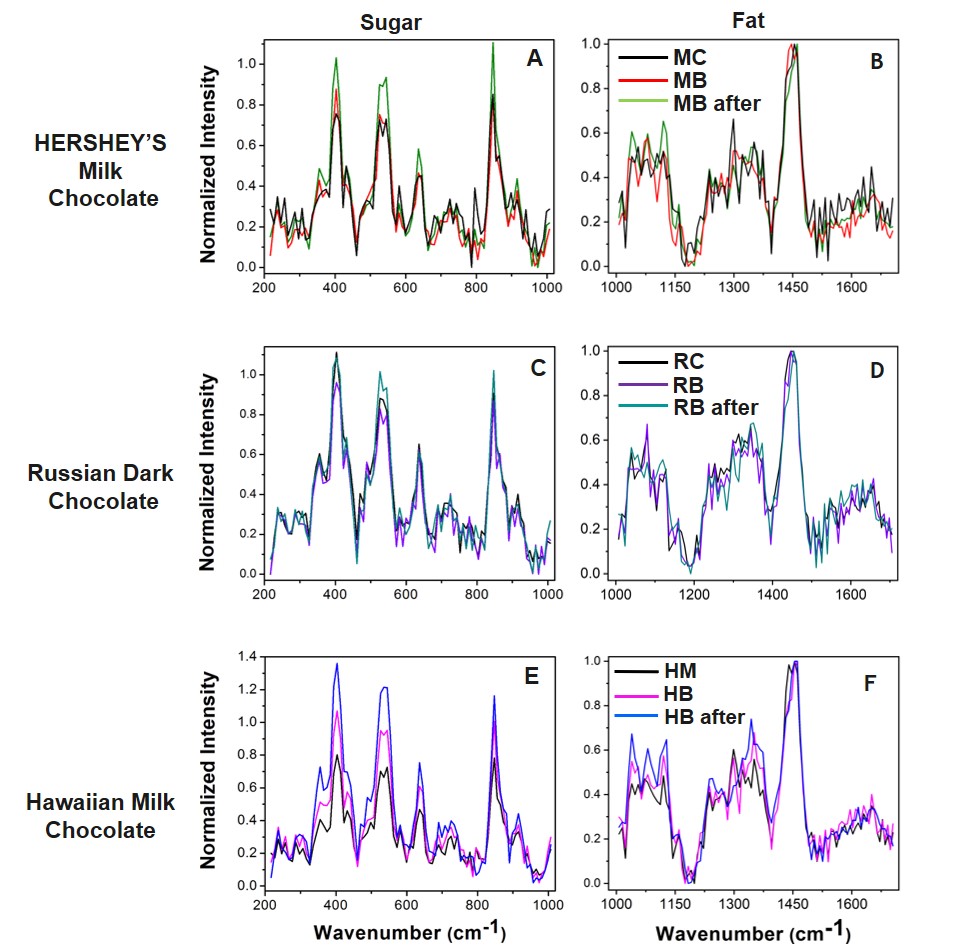


**Supplementary Fig. S4**. Normalized Raman spectra of laser bloom removal. The normalized spectra of the sugar (217 cm^-1^ to 1000 cm^-1^) and fat bands (1000 cm^-1^ to 1700 cm^-1^) are shown in the (**A**, **C**, **E**) and (**B,** **D**, **F**), respectively. The raw spectra were normalized by dividing the intensity of all bands by the highest cocoa butter band (1439 cm^-1^ to 1464 cm^-1^).


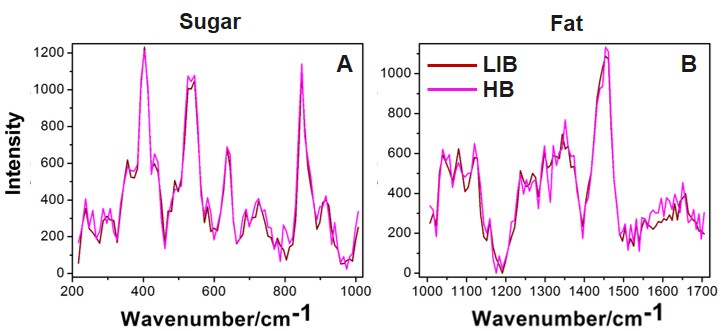


**Supplementary Fig. S5.** Non-normalized Raman spectra of laser-induced bloom. Non-normalized Raman spectra of the aging-induced bloom on the **HB** chocolate and of the laser-induced bloom (**LIB**) obtained from the white spot in **Fig. 4B**. The two spectra are almost identical, which suggests that the laser-induced bloom is chemically identical to the aging-induced bloom.


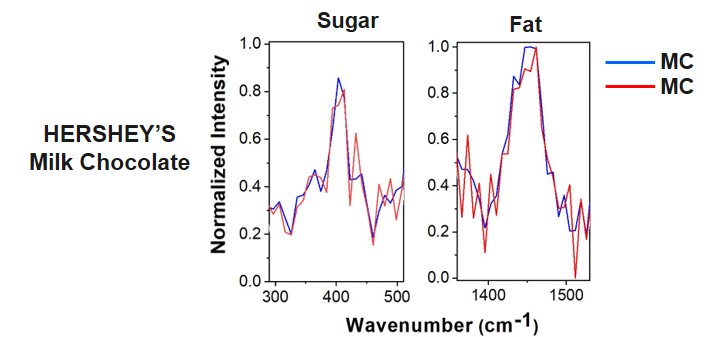


**Supplementary Fig. S6**. Two replicates of the normalized Raman spectra of HERSHEY’s milk chocolate (**MC**) in the sugar (300 cm^-1^ to 500 cm^-1^) and fat (1350 cm^-1^ to 1550 cm^-1^) spectral regions. The laser power was 350 mW with 5 s exposure time and 60 accumulations.


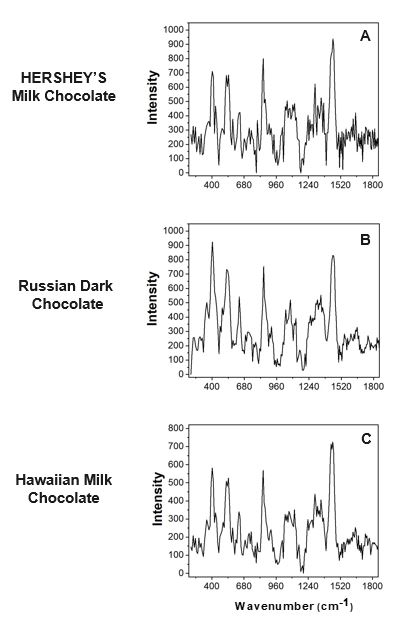


**Supplementary Fig. S7.** Non-normalized Raman spectra of the unbloomed **MC** (**A**), **RC** (**B**), and **HM** (**C**) chocolates in the spectral range from 217 cm^-1^ to 1800 cm^-1^. The laser power was 350 mW with 5 s exposure time and 60 accumulations.


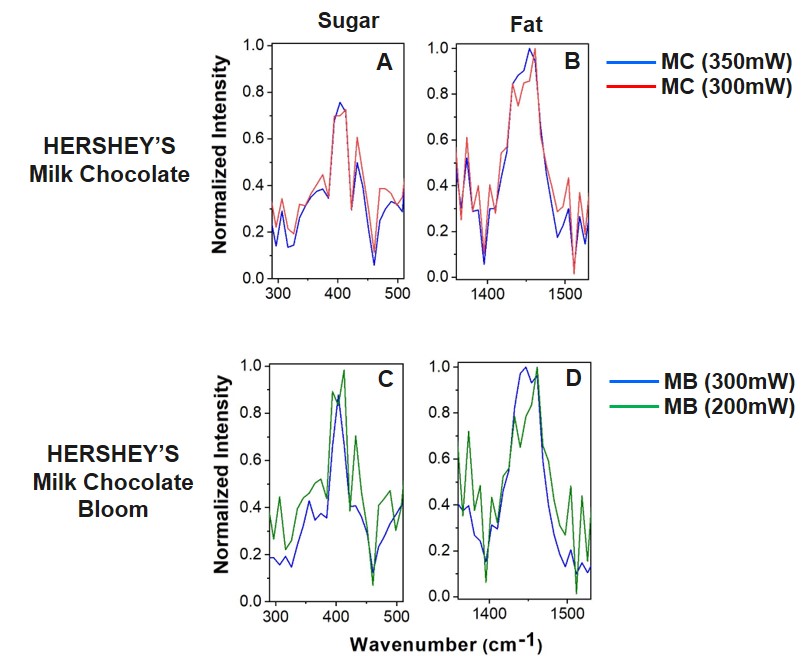


**Supplementary Fig. S8**. Normalized Raman spectra of HERSHEY’S milk chocolate and bloom using different laser powers. The normalized spectra of the sugar (217 cm^-1^ to 1000 cm^-1^) and fat bands (1000 cm^-1^ to 1700 cm^-1^) are shown in (**A**, **C**) and (**B,** **D**), respectively. The raw spectra were normalized by dividing the intensity of all bands by the highest cocoa butter band (1439 cm^-1^ to 1464 cm^-1^). The exposure time was set to 5 s with 60 accumulations.
